# Point/counterpoint: We should take the direction of blood pressure change into consideration for dynamic cerebral autoregulation quantification

**DOI:** 10.1177/0271678X221104868

**Published:** 2022-05-26

**Authors:** Lawrence Labrecque, Jonathan D Smirl, Yu-Chieh Tzeng, Patrice Brassard

**Affiliations:** 1 Department of Kinesiology, Faculty of Medicine, Université Laval, Québec, Canada; 2 Research Center of the Institut universitaire de cardiologie et de pneumologie de Québec, Québec, Canada; 3 Cerebrovascular Concussion Laboratory, Faculty of Kinesiology, University of Calgary, Calgary, Alberta, Canada; 4 Sport Injury Prevention Research Centre, Faculty of Kinesiology, University of Calgary, Calgary, Alberta, Canada; 5 Human Performance Laboratory, Faculty of Kinesiology, University of Calgary, Calgary, Alberta, Canada; 6 Hotchkiss Brain Institute, University of Calgary, Calgary, Alberta, Canada; 7 Integrated Concussion Research Program, University of Calgary, Calgary, Alberta, Canada; 8 Alberta Children’s Hospital Research Institute, University of Calgary, Calgary, Alberta, Canada; 9 Libin Cardiovascular Institute of Alberta, University of Calgary, Alberta, Canada; 10 Concussion Research Laboratory, Faculty of Health and Exercise Science, University of British Columbia, Kelowna, British Columbia, Canada; 11 Department of Surgery & Anesthesia, University of Otago, Wellington School of Medicine & Health Sciences, Wellington, New Zealand

**Keywords:** Asymmetry, cerebral pressure-flow relationship, directional sensitivity, dynamic cerebral autoregulation, mean arterial pressure

## Abstract

Accumulating evidence suggests asymmetrical responses of cerebral blood flow during large transient changes in mean arterial pressure. Specifically, the augmentation in cerebral blood flow is attenuated when mean arterial pressure acutely increases, compared with declines in cerebral blood flow when mean arterial pressure acutely decreases. However, common analytical tools to quantify dynamic cerebral autoregulation assume autoregulatory responses to be symmetric, which does not seem to be the case. Herein, we provide the rationale supporting the notion we need to consider the directional sensitivity of large and transient mean arterial pressure changes when characterizing dynamic cerebral autoregulation.

Cerebral autoregulation (CA) has commonly been described as the ability of the cerebral vasculature to react to steady-state (static CA) or transient (dynamic CA: dCA) arterial blood pressure (ABP) changes. The roles of these physiological responses are to maintain cerebral perfusion to meet the brain’s demand and minimize blood flow variations. Therefore, the extent of the vessels’ vasomotion (i.e. vasoconstriction or vasodilation) will be proportional to the ABP magnitude change, whether ABP increases or ABP decreases. The hypothesis behind the presence of directional sensitivity of the cerebral pressure-flow relationship resides in the role brain blood vessels play against ABP transient surges to protect the microcirculation from overperfusion. Therefore, it is thought dCA would be more efficient when ABP acutely increases in comparison with acute ABP decreases.

There is growing evidence of cerebral pressure-flow relationship directional sensitivity during large transient mean arterial pressure (MAP) oscillations. In healthy individuals, the first observation of a directional sensitivity was reported when comparing acute increases and decreases in ABP with drugs infusions.^1^ Using an autoregulatory gain calculation between middle cerebral artery mean blood velocity (MCAv) and MAP, Tzeng et al. demonstrated a smaller gain when MAP acutely increased compared with MAP decreases.^1^ We demonstrated a directional sensitivity of the cerebral pressure-flow relationship by examining changes in MCAv in response to transient increases and decreases in MAP induced by repeated squat-stands (i.e. a non-pharmacological model).^2–4^ Initially, these changes were calculated from a seated baseline,^2^ an approach that has been criticized. This calculation was further refined by using the largest concurrent MAP oscillations without an independent resting baseline, and by adjusting for time intervals.^3^ In addition to demonstrating a directional sensitivity in the MCA^3,4^ and in the posterior cerebral artery^4^ using this novel calculation, we suggested the hysteresis-like pattern is frequency-dependent (present during 0.10 Hz, but not 0.05 Hz, repeated squat-stands).^3,4^ A better autoregulatory capacity was also demonstrated during the squatting phase (transient increase in MAP) of repeated squat-stands using the autoregulatory index.^5^

Differences in cerebral sympathetic nervous activity (SNA) in response to transient MAP increases, compared with MAP decreases, could potentially explain this directional sensitivity of the cerebral pressure-flow relationship. Indeed, data in sleeping lambs indicate SNA recorded in the superior cervical ganglia is activated during acute ABP increases but not acute ABP decreases.^6^ The activation of cerebral SNA in response to an acute elevation in MAP would serve to protect the brain microcirculation from overperfusion and to reduce the risk of hemorrhagic stroke. Another potential mechanism underlying this hysteresis-like pattern could be a differing intrinsic myogenic activity when ABP increases, compared with ABP decreases. However, previous work suggests intrinsic myogenic activity influences dCA during oscillations below 0.07 Hz^7^ and calcium-channel blockade impairs dCA in humans during oscillatory lower body negative pressure at frequencies between 0.03 and 0.08 Hz.^8^

Of note, there are studies reporting the absence of asymmetry in the cerebral pressure-flow relationship^9^. Interestingly, Katsogridakis et al. used thigh-cuffs inflation and deflation to induce large transient MAP changes.^9^ This method has been shown to have extremely large variations in dCA metrics, even when tests are performed just minutes apart. Nonetheless, whether specific methods to induce significant MAP changes are partly responsible for the directional sensitivity of the pressure-flow relationship remains to be elucidated. Further research will thus be necessary to ensure this phenomenon is not method-dependent.

Directional sensitivity could be highly relevant to consider while studying CBF regulation in diverse populations. Since there are many physiological contexts (rapid eye movement sleep, high-intensity exercise) and clinical/pathological states (carotid stiffness, uncontrolled systemic hypertension, autonomic dysreflexia, anesthesia, etc.) involving acute and large changes in MAP, the direction of MAP has the potential of being an important variable to consider when examining dCA. We have recently reported that directional sensitivity of the cerebral pressure-flow relationship is present in sedentary and endurance-trained participants, but absent in resistance-trained individuals.^10^ Nonetheless, further studies are needed, as we still do not know whether this hysteresis-like pattern remains present in other physiological and pathological states. In addition, all studies focusing on the directional sensitivity of dCA to date included measures of cerebral blood velocity and MAP. The monitoring of volumetric cerebral blood flow and cerebral perfusion pressure (i.e. mean arterial pressure – intracranial pressure) in future studies is encouraged to provide more meaningful results not only in regards to the directional sensitivity of the cerebral pressure-flow relationship, but also for all the other metrics quantifying dCA.

It becomes clear there is asymmetrical sensitivity of the cerebral pressure-flow relationship during large transient MAP changes. We now need to better understand its underlying mechanisms and refine the current analytical tools to assess dCA, in order to eventually optimize the management of ABP in clinical populations. Replication of studies in various centers using different models of forced ABP oscillations (e.g. oscillatory lower body negative pressure) will also be crucial. The presence of asymmetrical sensitivity has important implications on quantification of dCA using standard analysis on forced ABP oscillations. For example, transfer function analysis, a common analytical tool to quantify dCA, assumes autoregulatory responses to be linear and symmetric, which is not the case. From now on, we consider we cannot solely use analytical methods that do not consider the direction of large transient MAP changes when characterizing dCA. It will remain crucial the scientific and medical community is aware of this important characteristic of the cerebrovasculature until a global change is undertaken by our community to take MAP direction into account in the assessment of dCA and acute manipulation of ABP within the clinical setting.
